# Influence of sigmoid plate and dura mater on vascular wall displacement, vibroacoustic/hydroacoustic sources characteristics, and frequency-loudness assessments of venous pulsatile tinnitus: A coupled-computational fluid dynamics study combining transcanal recording investigation

**DOI:** 10.3389/fbioe.2022.948230

**Published:** 2022-11-07

**Authors:** Xing Wang, Yue-Lin Hsieh, Xiaobing Xu, Wuqing Wang

**Affiliations:** ^1^ School of Mechanical and Automotive Engineering, Xiamen University of Technology, Xiamen, China; ^2^ Department of Otology and Skull Base Surgery, Eye Ear Nose & Throat Hospital, Fudan University, Shanghai, China; ^3^ NHC Key Laboratory of Hearing Medicine, Shanghai, China; ^4^ Department of Radiology, Eye and ENT Hospital, Fudan University, China

**Keywords:** pulsatile tinnitus, vibroacoustic analysis, hydroacoustic analysis, computational fluid dynamics, vascular displacement, dura mater, sigmoid plate dehiscence, transcanal recording

## Abstract

Investigations of pulsatile tinnitus (PT) caused by sigmoid sinus wall anomalies (SSWAs) using computational fluid dynamics (CFD) have recently increased in prevalence. However, accurate modeling of anatomical structures regarding sigmoid plate dehiscence and acoustic sources of PT remains lacking. This study incorporates coupled CFD techniques, micro-computed tomography, and scanning electron microscope to reveal the vibroacoustic and hydroacoustic sources and displacement characteristics of the transverse-sigmoid sinus system. Furthermore, the *in vivo* transcanal-recording technique combined with ipsilateral internal jugular vein compression was implemented to cross-reference the captured acoustic profile of PT with the calculated results. In this study, the transient state coupled CFD technique was used to calculate the vibroacoustic and hydroacoustic sources. The dehiscent sigmoid plate and periosteal dura mater were then reconstructed. The displacement characteristics and acoustic results were analyzed. The displacement of the vascular wall underneath the dehiscent area was 9.6 times larger than that of the sigmoid plate and 3,617 times smaller than that of the vascular wall without the overlying osseous structures. The peak amplitude of flow-induced vibroacoustic noise was 119.3 dB at 20.2 Hz measured at the transverse sinus. Within the observed 20–1,000 Hz frequency range, the largest peak amplitude of hydroacoustic noise was 80.0 dB at 20.2 Hz located at the jugular bulb region. The simulated results conformed with the *in vivo* acoustic profile which the major frequency of PT falls within 1,000 Hz. In conclusion, 1) the sigmoid plate and dura mater greatly impact vascular wall displacement, which should not be overlooked in CFD simulations. 2) By incorporating the transcanal recording technique with IJV compression test, the primary frequency of PT was found fluctuating below 1,000 Hz, which matches the frequency component simulated by the current CFD technique; amplitude-wise, however, the peak amplitude of *in vivo* pulse-synchronous somatosound measures approximately 10 dB, which is comparatively lesser than the CFD results and the subjectively perceived loudness of PT. Thus, the transmission pathway, intramastoid acoustic impedance/amplification effect, and the perceptive threshold of PT require further investigations to minimize the incidence of surgical failure.

## Introduction

Vascular pulsatile tinnitus (PT) is an abnormal sensation of vascular somatosound and/or forced vibroacoustic sound induced by blood flow impingement (Song et al., 2016; Eisenman et al., 2018; Mu et al., 2021a). This type of tinnitus is characterized by pulse-synchronous vascular bruits. Although subcategorized under objective tinnitus, venous PT is the most common type of vascular PT (Dong et al., 2015). The ipsilateral internal jugular vein (IJV) compression test helps indicate whence the pulse-synchronous noise arises, which this mechanistic characteristic has been used as a diagnostic criterion for indicating PT of venous etiologies (Hsieh et al., 2021b). In most cases with venous PT, sigmoid sinus wall anomalies (SSWAs), consisting of sigmoid sinus wall dehiscence and diverticulum, are the most common anatomical anomalies in patients with venous PT (Dong et al., 2015; Kline et al., 2020). Although there is no direct evidence, the formation of SSWAs has been attributed to the impact of transverse sinus flow and increased intracranial pressure (Huang et al., 2020; Ding et al., 2021; Qiu et al., 2021), which has been highlighted by recent growing clinical studies.

Sigmoid sinus cortical plate dehiscence is defined as the absence of an overlying bony plate that shields against the sigmoid sinus vascular wall (Eisenman, 2011). As dehiscence forms, PT arises under two theoretical assumptions: 1) the blood flow sound permeates through the defective sigmoid plate and causes PT—that is, the hydroacoustic source—and 2) the flow-induced displacement of the sigmoid sinus vascular wall gives rise to PT—that is, the vibroacoustic source (Eisenman et al., 2018; Hsieh et al., 2022a). Considering the fluid–structure interaction, the intraoperative placement of sensors and actuators inside the mastoid cavity or at the external auditory meatus has successfully captured the generated vascular sounds *in vivo*, indicating a high possibility of PT aerial transmittance dominating the bone conduction pathway (Kim et al., 2016; Hsieh et al., 2021b). However, these sensing applications have not yet been able to differentiate the generation sources of PT.

The coupled computational fluid dynamics (CFD) technique has successfully acquired the acoustic characteristics of vibroacoustic generation of noise secondary to sigmoid plate dehiscence (Tian et al., 2017; Mu et al., 2021a; Hsieh et al., 2022a). Prior to the realization of the fluid–structure interaction simulation, the *ad hoc* setting using the rigid vascular wall limited the possibility of investigating the generated sound originating from the forced vibration caused by the flow impact. Tian et al. (2017), Mu et al. (2021a).‘s pioneering works found that the vibroacoustic amplitude of PT ranged from 48.7 to 111.5 dB. Both groups suggested that wall pressure exerted on the sigmoid sinus vessel wall causes vascular displacement, which is a predominant underlying causative factor of vibroacoustic generation of PT; to that end, lowering the sinus wall pressure to reduce vibroacoustic noise generation can be pivotal to quiet PT (Tian et al., 2019a; Mu et al., 2021b). These findings provide a theoretical basis and indication for further surgical development.

Based on previous local anesthesia intraoperative discoveries, merely aiming to eliminate the vibration of the vascular wall by reconstructing the dehiscent region may not sufficiently preclude PT (Hsieh and Wang, 2020). Furthermore, a recent *in vivo* finding suggested that significant displacement differences in the osseous and vascular surfaces were found after the removal of the defective sigmoid plate and the dura mater underneath (Hsieh et al., 2022a). The bone and connective tissues overlying the sigmoid sinus vessel wall are microstructures that are often overlooked in computational settings but may improve our understanding of the biomechanics of venous PT associated with SSWAs.

Since the outcome of the CFD simulation is reliant on the boundary condition settings, this study aimed to establish a coupled CFD simulation based on actual human anatomical structures to investigate both hydroacoustic and vibroacoustic sources produced by intrasinus flow and the vibration of the sigmoid sinus vascular wall. The boundary conditions used in this study were significantly improved by integrating the data acquired from scanning electron microscope (SEM) and cadaveric specimens. Knowledge of the vibroacoustic and hydroacoustic characteristics inside the transverse-sigmoid sinus system yields a further understanding of the multiphysical perspective of PT, which can potentially benefit the future development of surgical methods.

## Materials and methods

### Patient clinical data

This study solicited a 25-year-old female patient with right-sided venous PT who sought medical advice at our PT clinic in December 2020. Her PT was eliminated during ipsilateral IJV compression monitored under Doppler ultrasound. The ipsilateral sigmoid sinus wall dehiscence was detected on the PT side on the high-resolution temporal computed tomography (CT). Sigmoid sinus wall dehiscence was defined as the absence of a bony plate overlying the sigmoid sinus contour with at least three consecutive horizontal 0.6-mm CT cuts without a vascular wall protruding into the mastoid air cells or mastoid cortex (Eisenman et al., 2018). Doppler ultrasound was performed to assess bilateral jugular hemodynamics.

All participants provided written informed consent. The experimental measurements were conducted in accordance with the principles of the Declaration of Helsinki. Ethical approval was obtained from the Ethical Committee of the Eye, Ear, Nose, and Throat Hospital in Shanghai, China.

### Surgical collection of dehiscent sigmoid plate for morphological analysis

**FIGURE 1:**
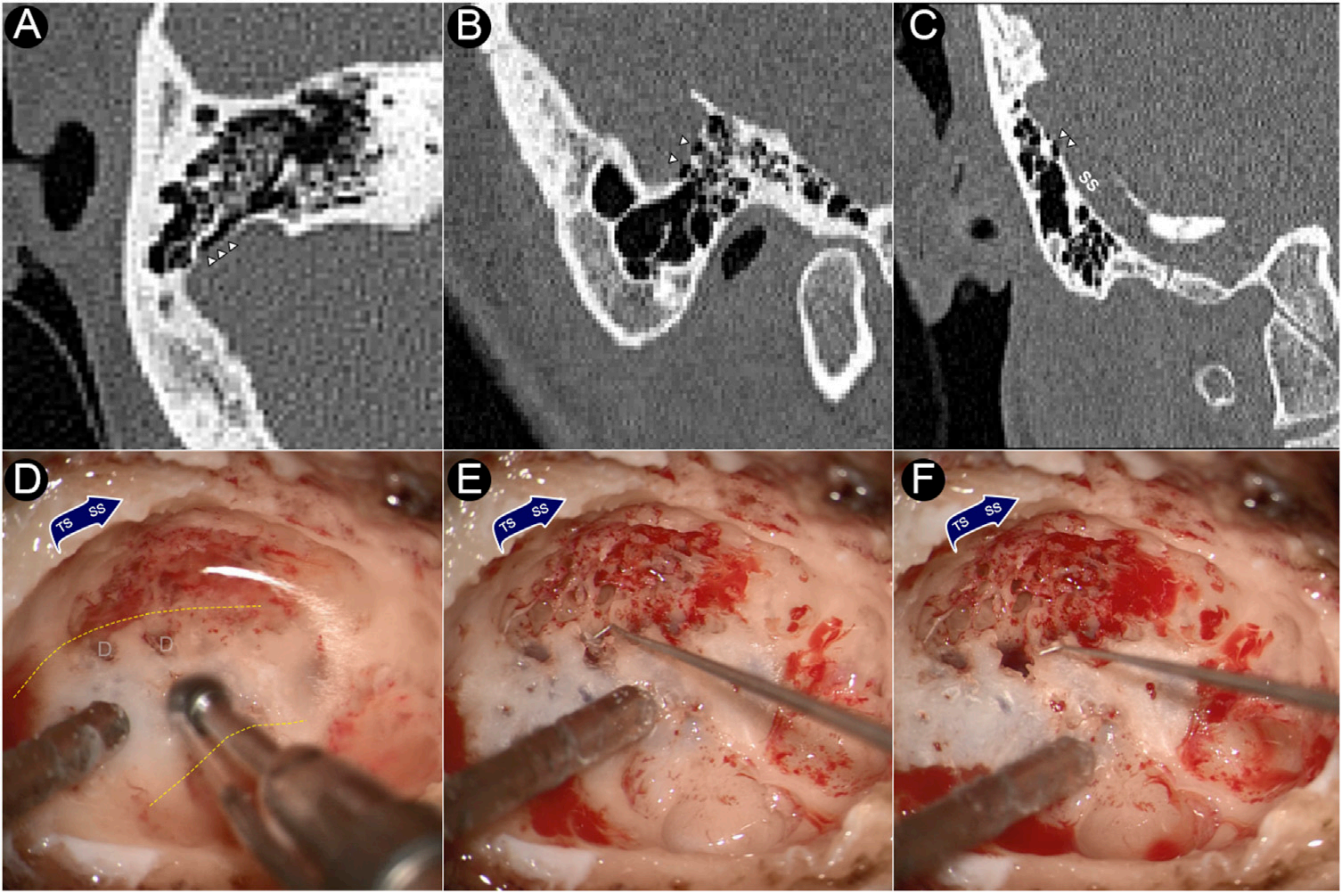
CT images and intraoperative photographs of the participant with sigmoid plate dehiscence. **(A)** Axial plane of dehiscence; **(B)** sagittal plane of dehiscence; **(C)** coronal plane of dehiscence. **(D)** Dehiscence exposed during skeletonization of sigmoid sinus. **(E)** Harvesting the dehiscent sigmoid plate. **(F)** After the removal of the dehiscent sigmoid plate. Triangles indicate the location of dehiscence. D represents dehiscence. Yellow dashed lines indicates the silhouette of the sigmoid sinus.

Intraoperative photographs and CT images of the dehiscence are shown in Figure 1. During the transtemporal surgery, the sigmoid sinus plate dehiscence was gradually exposed during the skeletonization of sigmoid sinus. After the mastoid air cells and osseous structures adjacent to the dehiscence were removed, the sigmoid sinus cortical plate and dehiscence was exposed. The bony structure overlying the vessel wall was deliberately preserved to prevent damage to the dehiscent bone tissue. The dehiscent plate was then harvested by separating the bone structure from the surrounding osseous wall and tissues underneath using a rectangular bone-burring pathway. The removed dehiscent sigmoid plate was immediately collected, placed in a fixative 4% paraformaldehyde solution for 24 h, and immersed in a solution of 75% alcohol for dehydration.

### Micro-computed tomography analysis of harvested sigmoid plate

**FIGURE 2:**
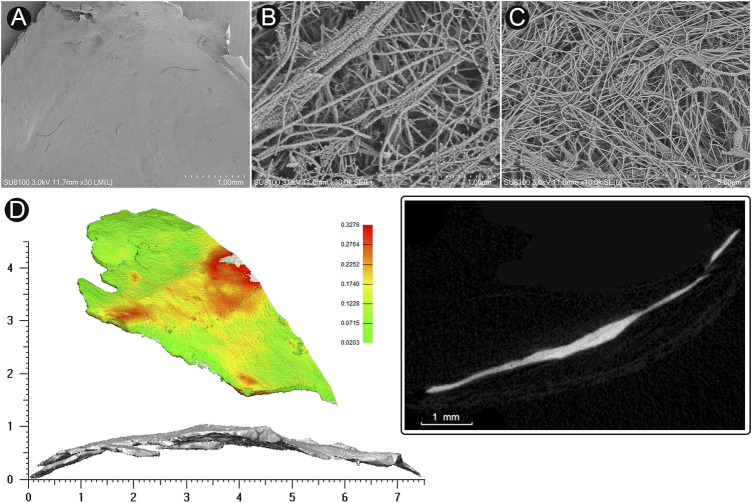
Microstructure and 3D reconstruction of dehiscent sigmoid plate. **(A)** SEM image of concave surface of the harvested sigmoid plate (magnification × 30). **(B)** (magnification × 30,000) and **(C)** (magnification × 10,000) SEM image of collagenic fibers of dura mater on the concave surface of the harvested sigmoid plate. **(D)** 3D reconstruction and μCT image of the harvested sigmoid plate.

To determine the thickness and microstructural component of the harvested dehiscent sigmoid plate, the harvested sigmoid plate from the participant was scanned using a SkyScan-1176 micro-CT (μCT) system (Bruker micro-CT, Belgium). The software packages Mimics 19.0 and 3-Matic 11.0 (Materialise, Belgium) were used to analyze the wall thickness of the bone plates. Figure 2 displays μCT images of the defective sigmoid plate overlying the sigmoid sinus wall.

### Cadaveric dura mater/vascular wall specimens and scanning electron microscope

**FIGURE 3:**
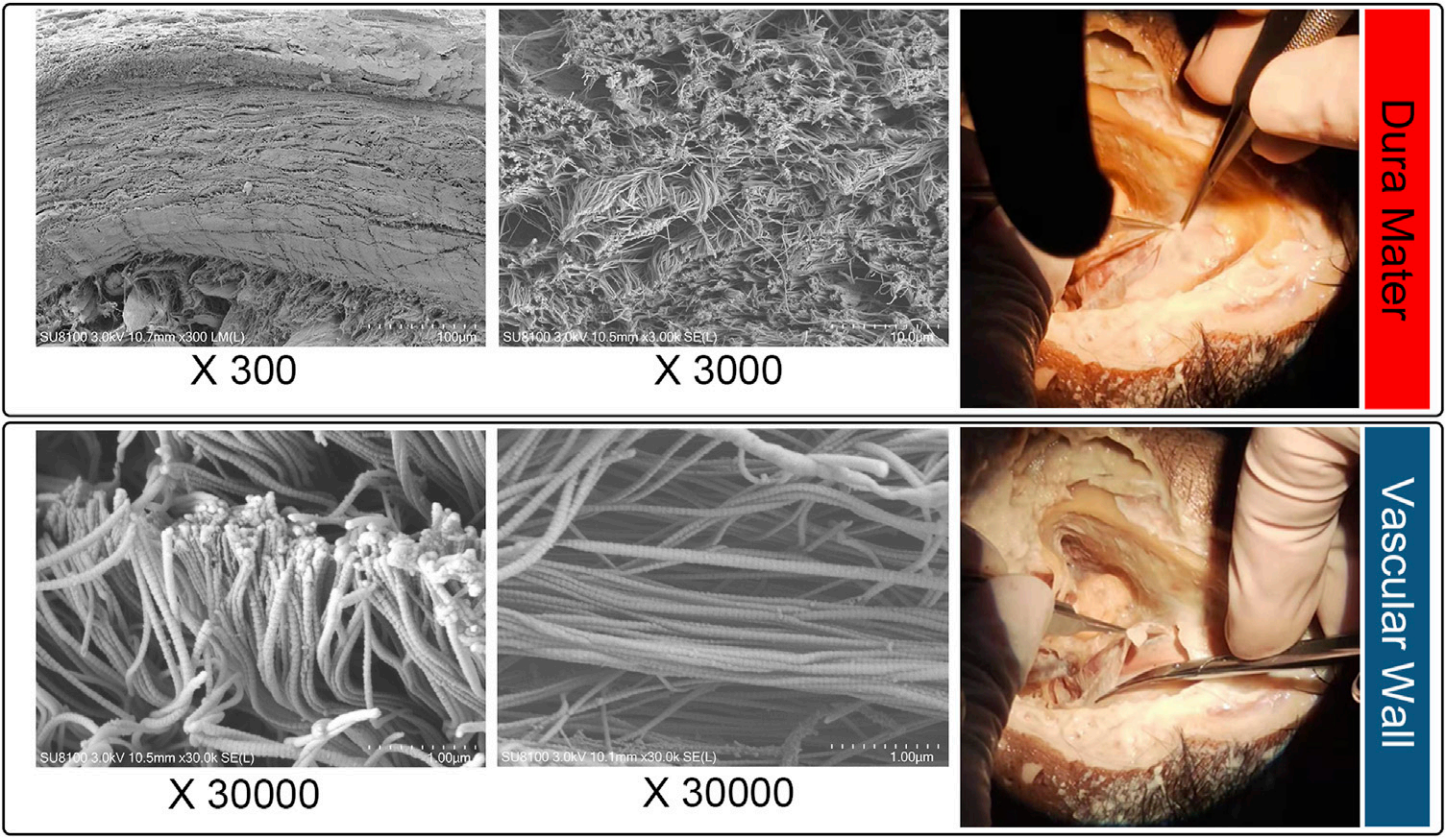
Microstructural examination of cadaveric specimens: dura mater (upper panel) and sigmoid sinus vascular wall (lower panel).

To thoroughly examine the thickness of dura mater and vascular wall, an ultrahigh-resolution field-emission scanning electron microscope (SEM) Regulus8100 (HITACHI, Japan) was used at 3.0 kV to estimate the thickness of the cadaveric dura mater/vascular wall specimens situated at the transverse-sigmoid junction acquired from the fresh frozen cadaveric head (Figure 3). The concave surface of the harvested dehiscent sigmoid plate was also examined to determine whether dura mater was present in the dehiscent area.

### Establishment of 3D finite-element models

**FIGURE 4:**
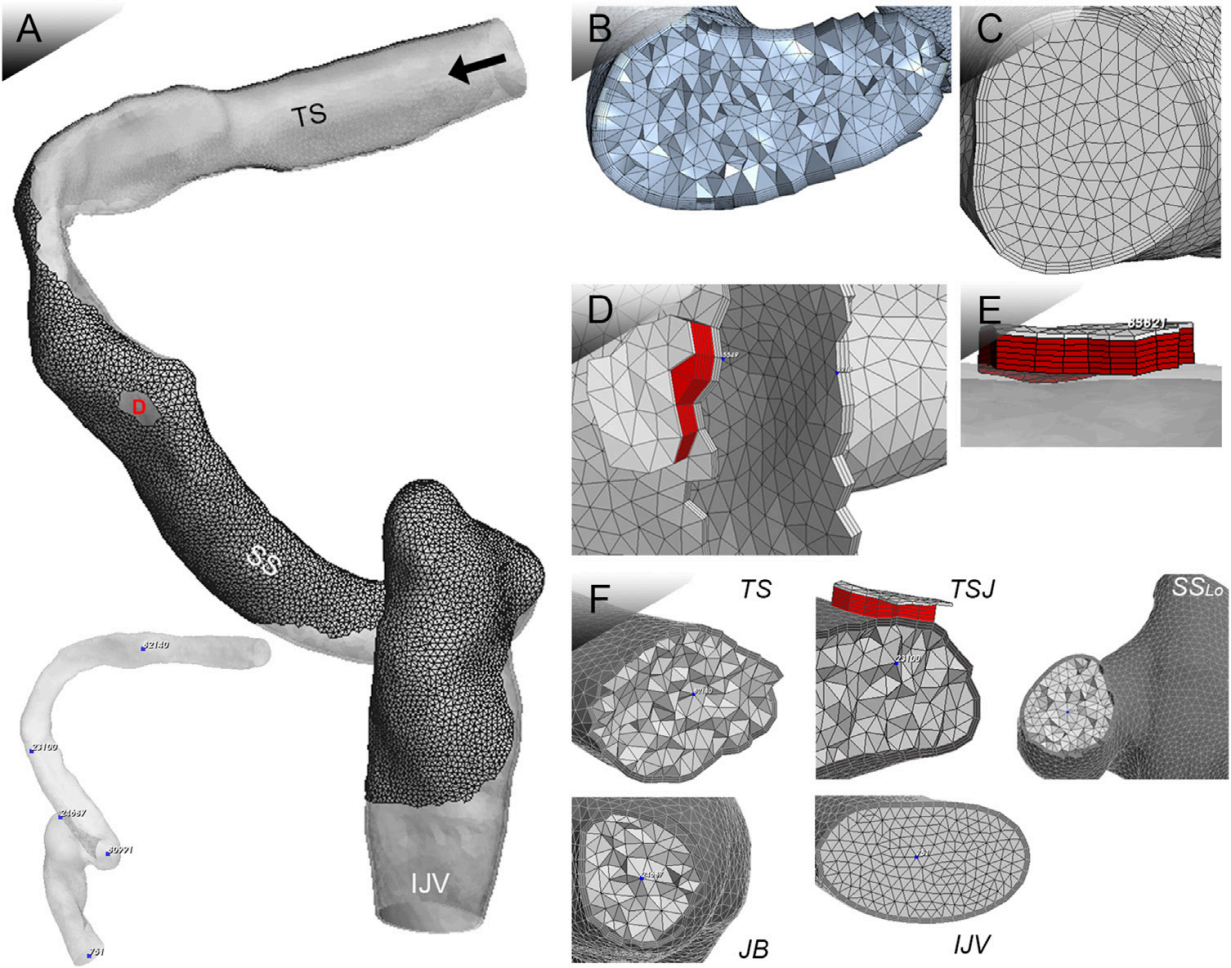
Patient-specific 3D FE model for coupled CFD studies. TS indicates transverse sinus; TSJ indicates transverse-sigmoid junction; SSLO indicates the lower curve of the sigmoid sinus; JB indicates jugular bulb; and IJV indicates internal jugular vein. Black arrow indicates flow direction. **(A)** Ipsilateral transverse-sigmoid sinus model, locations of virtual microphones, and the Doppler velocity spectrum. **(B)** Mesh of 3D geometric model for flow simulation. **(C)** Mesh of 3D geometric model for acoustic simulation. **(D)** and **(E)** Mesh of the dura mater and dehiscent sigmoid plate. **(F)** Cross-sectional displacement of locations of virtual microphones.

The 3D finite-element models were reconstructed based on patient-specific radiologic data using Mimics 19.0 and 3-matic 11.0 (Materialise, Belgium) (Figure 4). The ipsilateral transverse-sigmoid sinus was constructed, and the sinus branches were removed. A total of 1,050,377 with 10 surface layers and 274,517 tetrahedral elements were established on the identical vascular model were created for simulations of the flow and acoustic fields, respectively. Results of grid independence test are shown in Table 1. The average velocity of the IJV outflow (flow domain) and amplitude value at 250 Hz (acoustic domain) were selected as the criteria for generating a sufficient grid size and number. The relative error of both observed values were less than 5%, which were considered acceptable for this study.

**TABLE 1:** Fluid and acoustic mesh independence analysis.

Flow domain			
Number of mesh elements	Element size (mm)	Average internal jugular vein outflow velocity (m/s)	Relative error
463,696	0.5	1.5761e-01	0.246%
627,581	0.4	1.5772e-01	0.177%
1,050,377	0.3	1.5800e-01	

Acoustic domain			
Number of mesh elements	Element size (mm)	Amplitude at 250 Hz node 23,100 (dB)	Relative error
114,705	0.6	7.9604e2	0.009%
204,715	0.5	7.9609e2	0.002%
274,517	0.4	7.9611e2	

### Coupled computational fluid dynamics simulation

In the flow-field simulation, the outlet pressure was zero. The vascular wall was rigid. The flow velocity inlet was set based on patient-specific ipsilateral jugular flow velocity gauged using the Doppler ultrasound system MyLab Class C (Esaote, Genoa, Italy). The Navier–Stokes equations were solved using the transient laminar method Eqs 1,2:

∇∙u=0, (1)

$$\rho \frac{\partial u}{\partial t}+\rho u∙\nabla u=-\nabla p+\mu \nabla^{2}u,$$ (2)

where **u** is the velocity vector of the incompressible Newtonian blood flow, the blood density ρ is 1,050 kg/m^3^, and the dynamic viscosity μ is 0.00345 Pa s.

**FIGURE 5:**
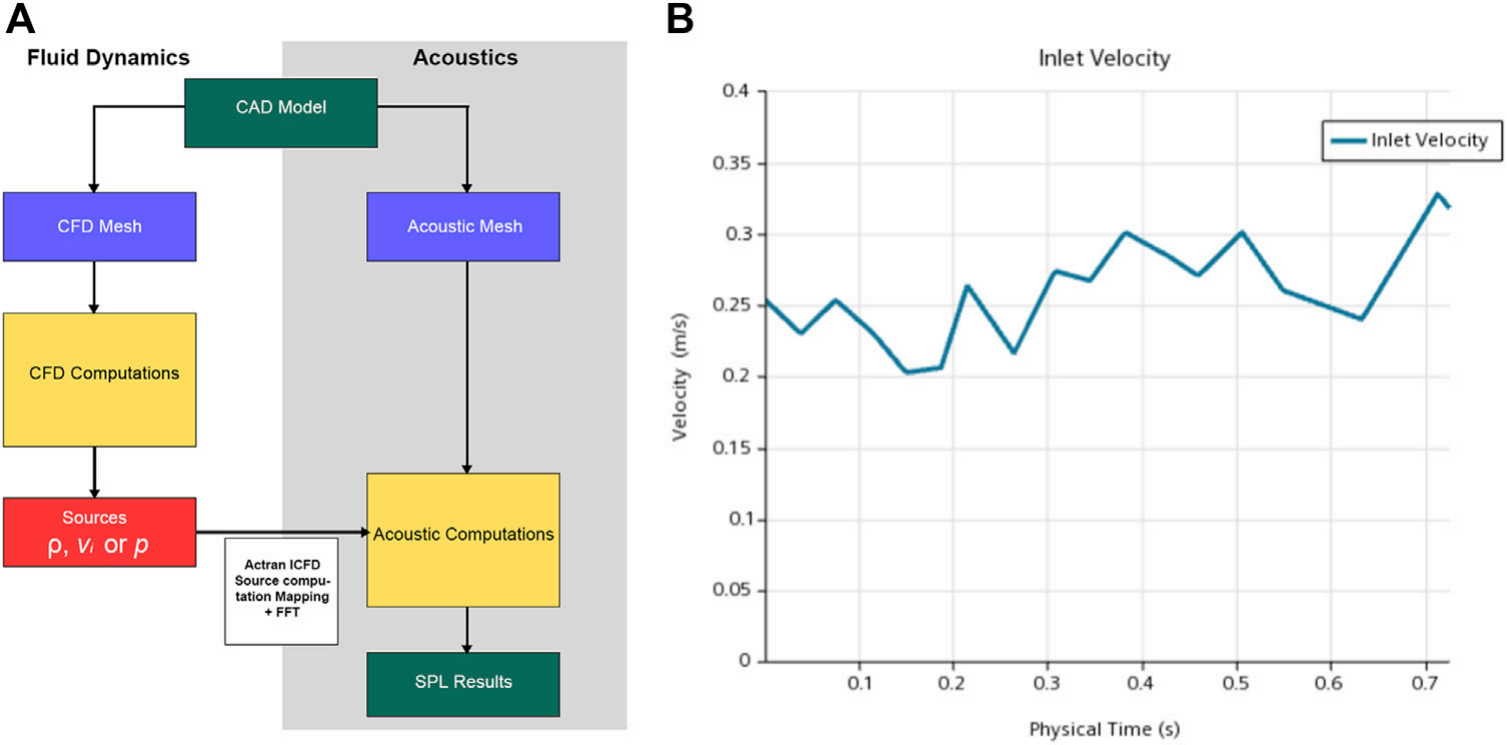
**(A)** Workflow of computational fluid dynamics (CFD) simulation. First, the pressure, density, and velocity fields output by the CFD solver were read; second, contributions according to the finite element method formulation were implemented; third, the contributions were projected on the acoustic mesh; fourth, the corresponding data were stored and displayed; and fifth, the quantities from the time to the frequency domain were transformed *via* Fourier transform. **(B)** The velocity inlet set for computational simulation.

The one-way coupled simulation comprised five major steps (see Figure 5 for the complete simulation workflow and the figure caption for the description). A quadrupole sound source was set up for the transformation of the pressure fluctuation and flow velocity. For incompressible CFD computations, only the velocity field was required. The observed frequency range was 0–1 kHz. The reference pressure was set at 20 μPa. Three pulsatory cycles were calculated, and the second pulsatory cycle was chosen for data presentation.

For the computation of wall pressure fluctuations, the hydrodynamic and the acoustic wall pressure fluctuation were extracted and computed from the unsteady CFD results. To investigate the displacement of the vascular walls, the regional vascular walls at the dehiscent area and regions without overlying osseous structures were set as vibrating walls inside the acoustic simulation domain. The vascular wall leaning against the temporal bone was rigid. To set the dura mater and sigmoid plate, we designed additional moving layers above the outer surface of the vascular wall at a specific dehiscent area. The mechanical properties of the temporal dura mater, sigmoid plate, and sigmoid sinus vascular wall are listed in Table 2 (Peterson and Dechow, 2003; Persson et al., 2010; Tian et al., 2017). The retroaction effect between the fluid/structure is neglected. Starting from the momentum equation in the fluid Eq. 3:

$$\frac{\partial \rho v_{i}}{\partial t}+\frac{\partial \rho v_{i}v_{j}}{\partial x_{j}}=\frac{\partial \sigma_{ij}}{\delta x_{j}},$$ (3)

where the integral formation of the equation over a flow domain was Eq. 4:

$$\underset{V}{\int }\frac{\partial \rho v_{i}}{\partial t}dv+\underset{V}{\int }\frac{\partial \rho v_{i}v_{j}}{\partial x_{j}}dv=\underset{V}{\int }\frac{\partial \sigma_{ij}}{\partial x_{j}}dv,$$ (4)

where the right-hand side of Eq. 4 indicates the retrieved exterior forces interacting on the boundaries of the flow medium, which can be rewritten as Eq. 5:

$$\underset{V}{\int }\frac{\partial \sigma_{ij}}{\partial x_{j}}dv=\oint_{S}\sigma_{ij}n_{j}ds$$ (5)

with Eq. 6

$$\sigma_{ij}=-p\delta_{ij}+\tau_{ij},$$ (6)

which *p* is the fluid pressure and $\tau_{ij}$ is the viscous stress tensor. $n_{j}$ is the normal direction of ds . ds is the surface of dv . For imcompressible flow $div\left(\vec{u}\right)=0$ (Oberai et al., 2000).

**TABLE 2:** Mechanical properties of components used for boundary condition settings.

	Thickness (mm)	Density (kg/m^3^)	Poisson’s ratio	Young’s modulus (MPa)
Harvested sigmoid plate	0.1	1868	0.3	12,000
Dura mater	0.7	1,174	0.45	70.0
Vascular wall	0.3	1,050	0.3	1.26

In the hydroacoustic simulation, variational formulations of Lighthill’s analogy were implemented, starting with Lighthill’s equation based on (Oberai et al., 2000) Eqs 7,8:

$$\frac{\partial^{2}}{\partial t^{2}}\left(\rho -\rho_{0}\right)-c_{0}^{2}\frac{\partial^{2}}{\partial x_{i}\partial x_{j}}\left(\rho -\rho_{0}\right)=\frac{\partial^{2}T_{ij}}{\partial x_{i}\partial x_{j}},$$ (7)

with

$$T_{ij}=\rho u_{i}u_{j}+\delta_{ij}\left(\left(p-p_{0}\right)-c_{c}^{2}\left(\rho -\rho_{0}\right)\right)-\tau_{ij},$$ (8)

where p and ρ are pressure and density, respectively. $\rho_{0}$ was the reference value. $\tau_{ij}$ is the viscous stress tensor. $c_{0}$ is the reference sound velocity, and $T_{ij}$ , $u_{i}$ , and $u_{j}$ are the Lighthill tensor and fluid velocity components, respectively. The variational formulation of Lighthill’s analogy was now rewritten as Eq. 9 after the strong variational formulation of Eq. 7 and integration by parts along spatial derivatives following Green’s theorem ( $\rho_{a}=\rho -\rho_{0}$ ):

$$\underset{\Omega }{\int }\left(\frac{\partial^{2}\rho_{a}}{\partial t^{2}}\delta \rho +c_{c}^{2}\frac{\partial \rho_{a}}{\partial x_{i}}\frac{\partial \rho_{a}}{\partial x_{i}}\right)dx=-\underset{\Omega }{\int }\frac{\partial T_{ij}}{\partial x_{j}}\frac{\partial \delta \rho }{\partial x_{i}}dx+\underset{\partial \Omega =\Gamma }{\int }\frac{\partial \sum_{ij}}{\partial x_{j}}n_{i}\delta \rho d\Gamma ,$$ (9)

where δρ is the test function, Ω is the computational domain, and $\sum_{ij}$ is Eq. 10:

$$\sum_{ij}=\rho u_{i}u_{j}+\left(p-p_{0}\right)\delta_{ij}-\tau_{ij},$$ (10)

The source term volume contribution $\int_{\Omega }\frac{i}{\rho_{0}\omega }\frac{\partial \delta \psi }{\partial x_{i}}\frac{\partial }{\partial x_{i}}F\left(\rho u_{i}u_{j}\right)d\Omega$ volume integral using the unsteady velocity and density fields saved in the CFD files that relate to the acoustic mesh ( δψ ) were computed. F is $\frac{\delta \psi }{i\omega }\frac{\partial }{\partial x_{i}}\left(c^{2}\rho \delta_{ij}+T_{ij}\right)n_{i}$ . Hydroacoustic simulation has been previously described by our recent in detail (Gao et al., 2022). The variational formulation of Lighthill’s analogy was integrated by parts and applied to the right-hand side, where the surface contribution was derived from an integral over Eq. 11:

$$-\int_{\Gamma }\frac{1}{\rho }F\left(\rho u_{i}n_{i}\right)\delta \psi d\Gamma .$$ (11)

For rigid walls, a zero normal velocity condition was applied, where the boundary integral disappeared; for vibrating walls, the normal forces distributed over the surface were calculated. The audio file of the computed results (Supplementary File S1) was extracted and rated by the participant.

### Data acquisition on different anatomical locations

Displacement data of the sigmoid plate (node 83,821), the internal surface of the sigmoid sinus vessel wall underneath the dehiscence (node 5,549), and the brain (medial) side internal surface of the sigmoid sinus vessel wall (node 5,216) were chosen.

To acquire vibroacoustic and hydroacoustic sound sources, the virtual microphone was set at the center of the vascular lumen: 1) transverse sinus (node 82,140); 2) transverse-sigmoid junction (node 23,100); 3) lower curve of the sigmoid sinus (node 60,991); 4) jugular bulb (node 24,687); and 5) upper IJV (node 751).

### Objective external ear canal recording in junction with internal jugular vein compression and Doppler ultrasound recording of pulsatile tinnitus

The trans-canal recording method was first quantitatively and qualitatively introduced by Song et al. (2016). An omni-directional lavalier electric condenser microphone BY-M1 Pro (Shenzhen Jiayz Industrial., Ltd., China) with frequency range 65 Hz–18 KHz and sensitivity −30 dB+/−3 dB re = 1 V/Pa, 1 kHz. The participant sat in a soundproof booth with the microphone probe inserted into the ipsilesional external auditory meatus. The signals were recorded using Adobe Audition cc 2020 (Adobe Inc.). Compression of the ipsilateral IJV was performed during the recording session. Sonification of the acoustic profiles and short-time Fourier transformation analysis were executed equivalent to our previous setups (Hsieh et al., 2021a; Hsieh et al., 2021b; Hsieh et al., 2022b) using MATLAB (MathWorks). The original captured acoustics before and after IJV compression were compared to showcase the relative amplitude reduction with and without PT under the same recording environment and post-analysis methods. Supplementary File S2 (without IJV compression) and Supplementary File S3 (IJV compressed) are the outcomes of transcanal recordings. The recording of Doppler ultrasound hemoacoustics (hydroacoustic source of blood flow motion) was carried out identical to our previous methods (Hsieh et al., 2021a) (Supplementary File S4).

### Subjective psychoacoustic matching

Frequency matching was performed using a Madsen Astera audiometer (Natus Medical Denmark ApS, Taastrup, Denmark) and TDH-39P headphones (Telephonics Ltd., Farmingdale, NY, United States). The interaural difference in the hearing threshold of the participant was below 10 dBHL. The participant was instructed to compare the given narrow-band noises at each center frequency of 125, 250, 500, and 750 Hz and 1, 1.5, 2, and 3 kHz sequentially in the contralateral ear, where the likeness of PT was rated after the stimuli were given twice. The loudness was matched using 125 Hz 1/3 octave narrow-band noise, increasing from the hearing threshold level in 1-dB steps. The participant was asked to rate likeness using a 0 –10 scale among all subjective tests and the audio files of the *in vivo* recorded pulse-synchronous somatosounds. To maintain the consistency of the pulsatory sound, nine pulsatory cycles were performed using the representative CFD calculated results from the transverse-sigmoid junction (node 23,100).

## Results

### Morphologies and characteristics of harvested sigmoid plate and cadaveric dura mater and vascular wall specimens

The area of the harvested sigmoid plate was approximately 4.5 × 7.0 mm^2^. The median thickness of the harvested sigmoid plate was 0.12 (0.08/0.17) mm. The surface area was 40.3 mm^2^. The bone volume/total volume (percent bone volume) ratio was 81.3%. The bone surface/volume ratio was 0.268 (1/pixel). The bone surface density was 0.218 1/pixel. The trabecular thickness was 13.0884. The number was 0.062 1/pixel. The total porosity was 18.67%. Under SEM examination, layers of the periosteal dura mater were located on the concave surface of the harvested sigmoid plate with collagenic fibers (Figure 2). Collagenic fibers of the dura mater and transverse-sigmoid junction vessel walls moved together and were mutually fixated. SEM photographs of the collagenic architecture of the outermost dura mater and the transverse-sigmoid junction vessel wall are shown in Figure 3.

### Hemodynamic outcomes

**FIGURE 6:**
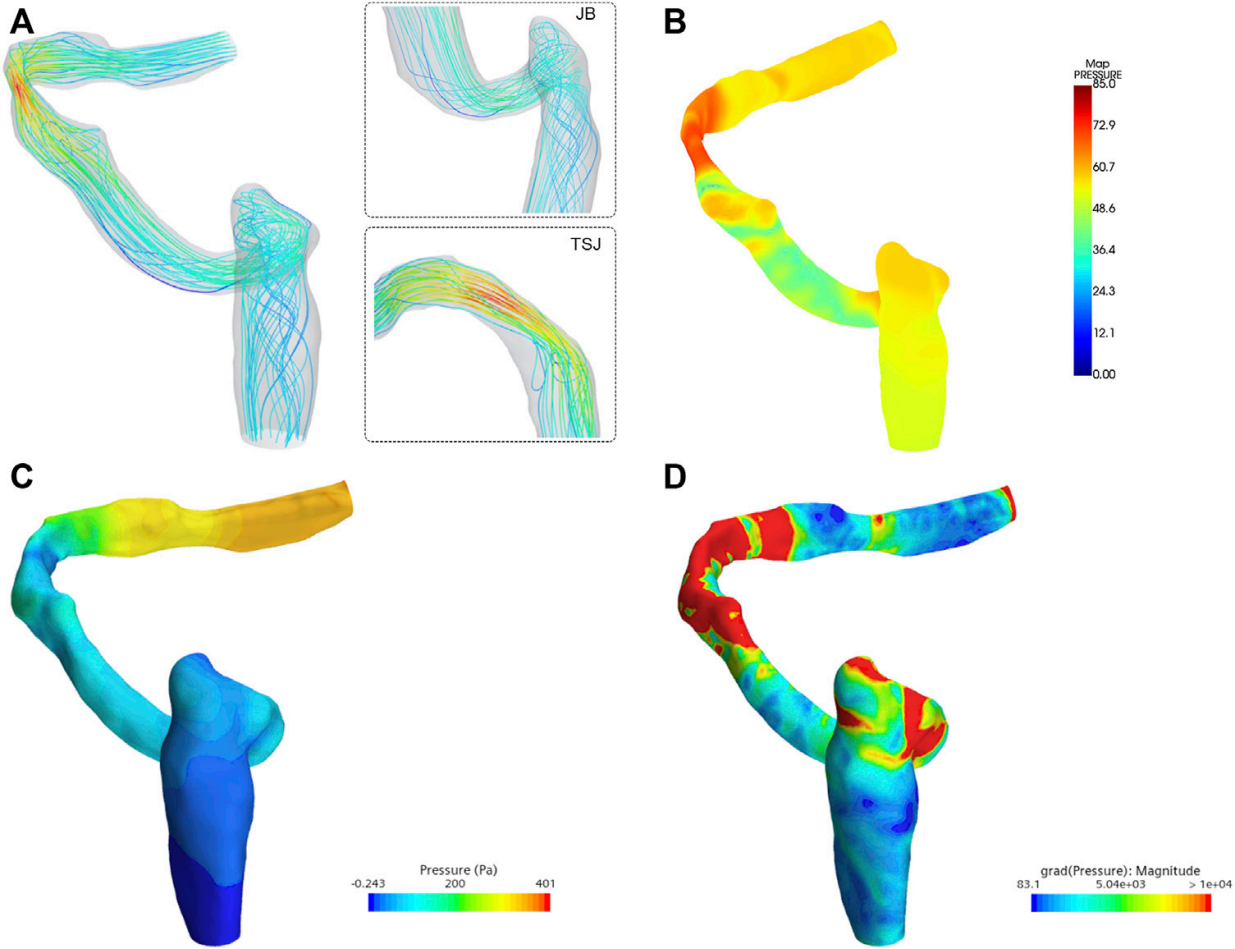
Hemodynamic characteristics of the flow field. TSJ indicates transverse-sigmoid sinus. JB indicates jugular bulb. **(A)** Flow velocity and streamline, **(B)** visualization of the acoustic field (unit: dB), **(C)** wall pressure distribution, and **(D)** pressure gradient inside the ipsilateral transverse-sigmoid sinus (unit: Pa/m).

The average velocity and peak velocity of the transverse-sigmoid sinus flow field were 0.198 m/s and 0.745 m/s, respectively. The minimum and maximum wall pressures of the transverse-sigmoid sinus system was −0.2 and 401 Pa. The largest pressure monitored measured at the location of sigmoid plate was 84.0 Pa. The average pressure gradient from the inlet to the outlet was 5.72e+3 Pa/m. A visualization of the hemodynamics is shown in Figure 6.

### Displacement characteristics of anatomical structures

**FIGURE 7:**
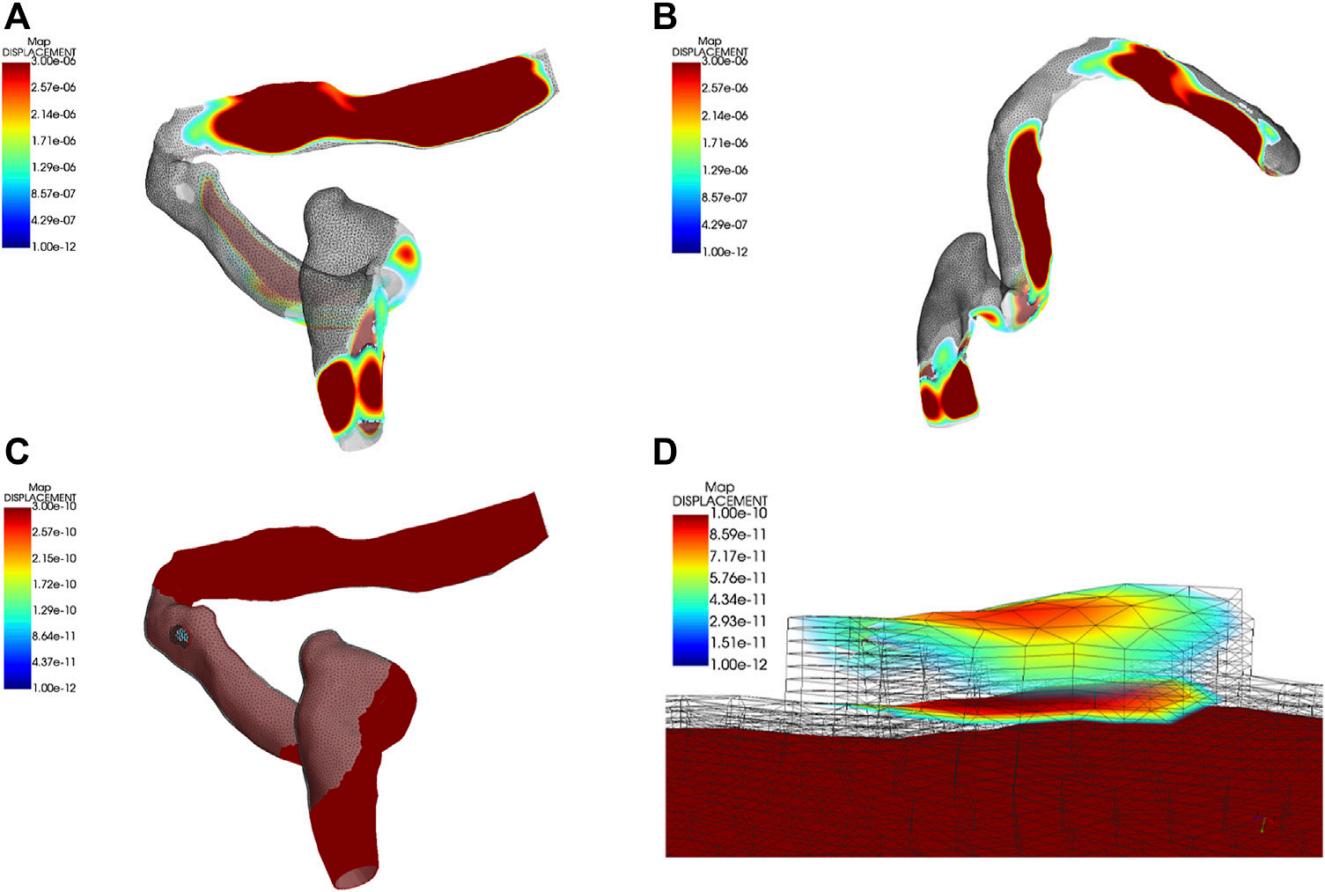
Displacement characteristics ipsilateral transverse-sigmoid sinus system. Units in graphs A to D are meters. **(A)** and **(B)** Visualization of the displacement of the ipsilateral transverse-sigmoid sinus. **(C)** and **(D)** Visualization of the dehiscent sigmoid plate.

**FIGURE 8:**
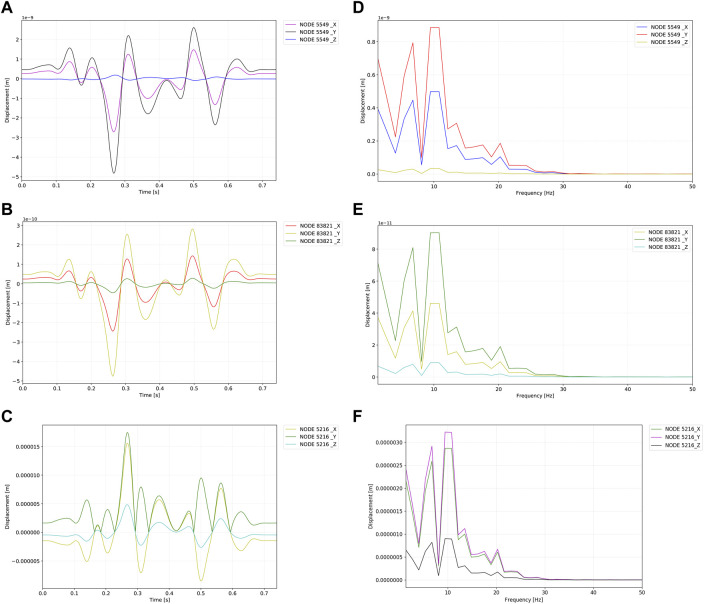
Displacement profiles of the observed nodes. Units in graphs A to F are meters. **(A)** Time–displacement of the internal surface of the sigmoid sinus vessel wall underneath dehiscence (node 5,549). **(B)** Time–displacement of the sigmoid plate underneath dehiscence (node 83,821). **(C)** Time–displacement of the brain side internal surface of the sigmoid sinus vessel wall (node 5,216). **(D)** Frequency–displacement of the internal surface of the sigmoid sinus vessel wall underneath dehiscence (node 5,549). **(E)** Frequency–displacement of the sigmoid plate underneath dehiscence (node 83,821). **(F)** Time–displacement of the brain side internal surface of the sigmoid sinus vessel wall (node 5,216).

The outcomes and visualization of the displacement are shown in Figures 7, 8. The median and root mean square (RMS) displacement measured at the sigmoid plate (node 83,821) were 4.3e-11 (−5.6e-11/6.1e-11) m and 1.3e−11 m, in which the largest displacement was 7.3e−10 m within a pulsatory cycle appeared from 0.26 to 0.31 s. The peak displacements of all observed nodes were found in the *Y* direction. Compared to the displacement of the sigmoid plate, the largest displacement at the internal surface of the sigmoid sinus vascular wall underneath the bony dehiscence (node 5,549) was 9.6 times larger than that of the sigmoid plate (node 83,821). The median and RMS displacements gauged at node 5,549 were 4.5e−10 (-6.2e-10/7.0e-10) m and 5.1e−11 m, respectively, and the largest displacement was 7.0e−9 m. Because the vascular wall attached to the soft tissues was not restricted, the displacement magnitude at the nonrestricted vascular wall (node 5,216) was 3,617 times larger than that at node 5,549. The median and RMS displacements at node 5,216 were −1.61e−6 (−2.4e-6/2.2e−6) m and 1.3e−6 m, respectively, and the largest displacement was 2.54e−05 m. The magnitude of the displacement decreased significantly after 20 Hz among all measured nodes.

### Computational fluid dynamics outcomes of vibroacoustic and hydroacoustic noise productions

**FIGURE 9:**
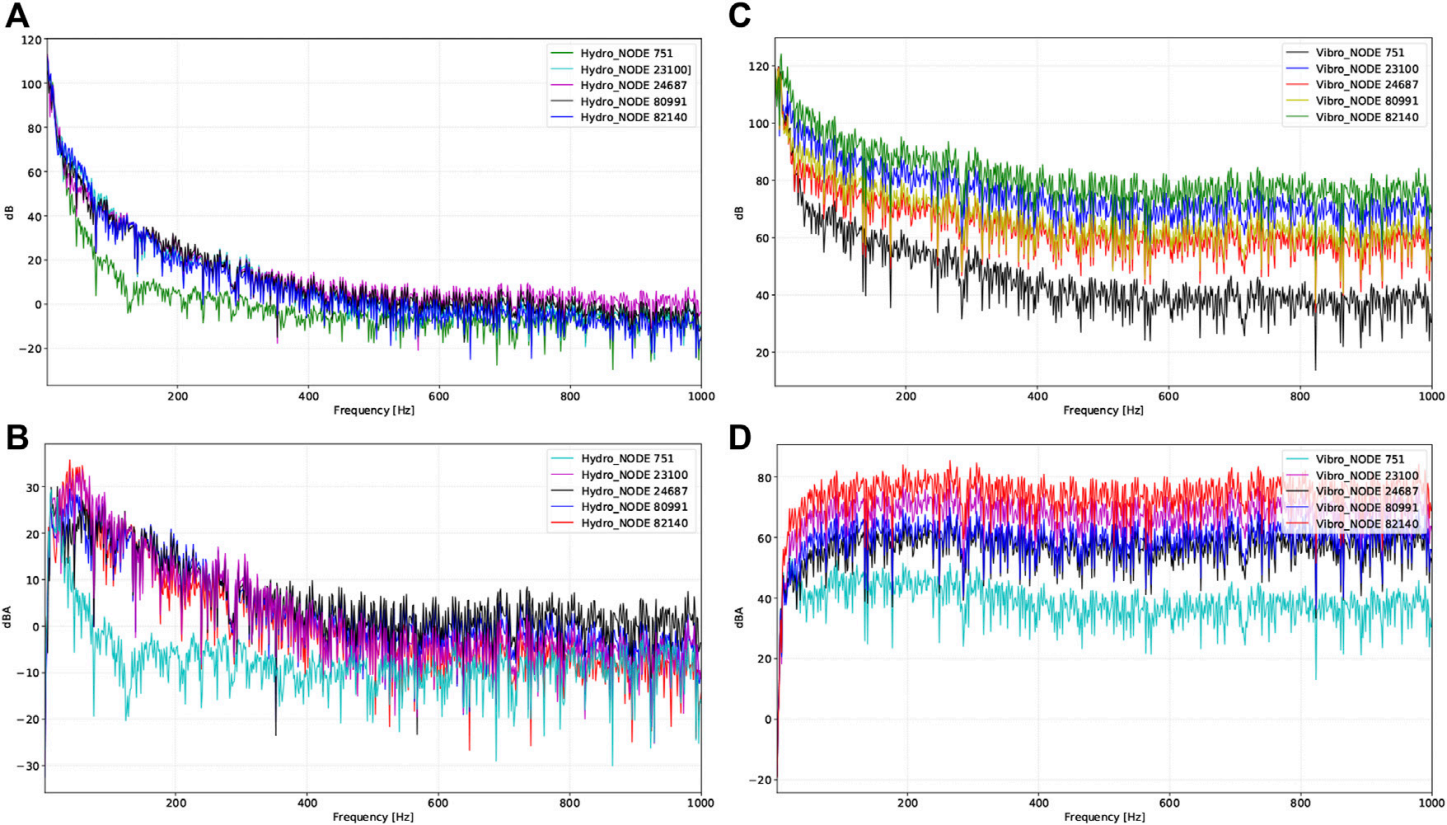
Vibroacoustic and hydroacoustic characteristics and visualization of acoustic field captured by different locations of virtual microphones. Representative nodes are 1) transverse sinus (node 82,140), 2) transverse-sigmoid junction (node 23,100), 3) lower curve of sigmoid sinus (node 60,991), 4) jugular bulb (node 24,687), and 5) upper internal jugular vein (node 751). **(A)** Hydroacoustic results at different locations in dB. **(B)** Hydroacoustic results at different locations in dBA. **(C)** Vibroacoustic results at different locations in dB. **(D)** Vibroacoustic results at different locations in dBA.

Results of the vibroacoustic and hydroacoustic production at disparate locations are shown in Figure 9 and Table 3. Based on the results of the chosen nodes, the RMS amplitude of vibroacoustic sound generation was highest at the transverse sinus and decreased by 40.7% with a 33.4 dB gradient at the upper internal jugular vein. The peak amplitude of flow-induced vibroacoustic noise was 119.3 dB at 20.2 Hz measured at the transverse sinus (node 82,140). Within the observed 20–1,000 Hz frequency range, the largest peak amplitude of hydroacoustic noise was 80.0 dB at 20.2 Hz located at the jugular bulb region (node 24,687). Within 20–1,000 Hz, the RMS amplitude of the vibroacoustic source was 65.7 dB; the RMS amplitude of the hydroacoustic noise source was 24.2 dB at the transverse-sigmoid junction. The difference of hydroacoustic RMS amplitude inside the transverse-sigmoid junction was less than 1.5 dB.

**TABLE 3:** Results of acoustic profiles measured at different anatomical locations.

Anatomical locations		Transverse sinus	Transverse-sigmoid junction	Lower curve of sigmoid sinus	Jugular bulb	Upper internal jugular vein
Position of virtual microphones (Node Numbers)		82,140	23,100	80,991	24,687	751
VIBRO	Peak amplitude^a^ (dB/dBA)	119.3/85.4	111.2/78.7	102.0/69.8	103.4/67.7	106.2/56.1
	RMS amplitude^a^ (dB/dBA)	82.0/74.1	75.4/67.4	67.5/59.4	64.4/56.2	48.6/56.1
	Frequency at peak amplitude (Hz_dB_/Hz_dBA_)	20.2/264.8	20.2/20.2	20.2/264.8	20.2/264.8	20.2/264.8
HYDRO	Peak amplitude^a^ (dB/dBA)	74.3/35.8	76.3/33.7	76.5/29.5	80.0/30.0	77.7/27.7
	RMS amplitude^a^ (dB/dBA)	23.7/11.7	24.2/11.8	23.2/10.5	23.2/10.2	18.5/10.5
	Frequency at peak amplitude^b^ (Hz_dB_/Hz_dBA_)	20.2/39.1	20.2/58.1	20.2/39.10	20.2/20.2	20.2/20.2

^a^ indicates the amplitude of the observed frequency within 20–1,000 Hz. VIBRO and HYDRO indicates parameters of vibroacoustic and hydroacoustic noise.

^b^ Hz_dB_/Hz_dBA_ indicates the frequency at peak amplitude (dB)/(dBA).

### Characteristics and ratings of *in vivo* trans-canal recording, *in vivo* doppler, computational fluid dynamics calculated audio, and psychoacoustic matching

**FIGURE 10:**
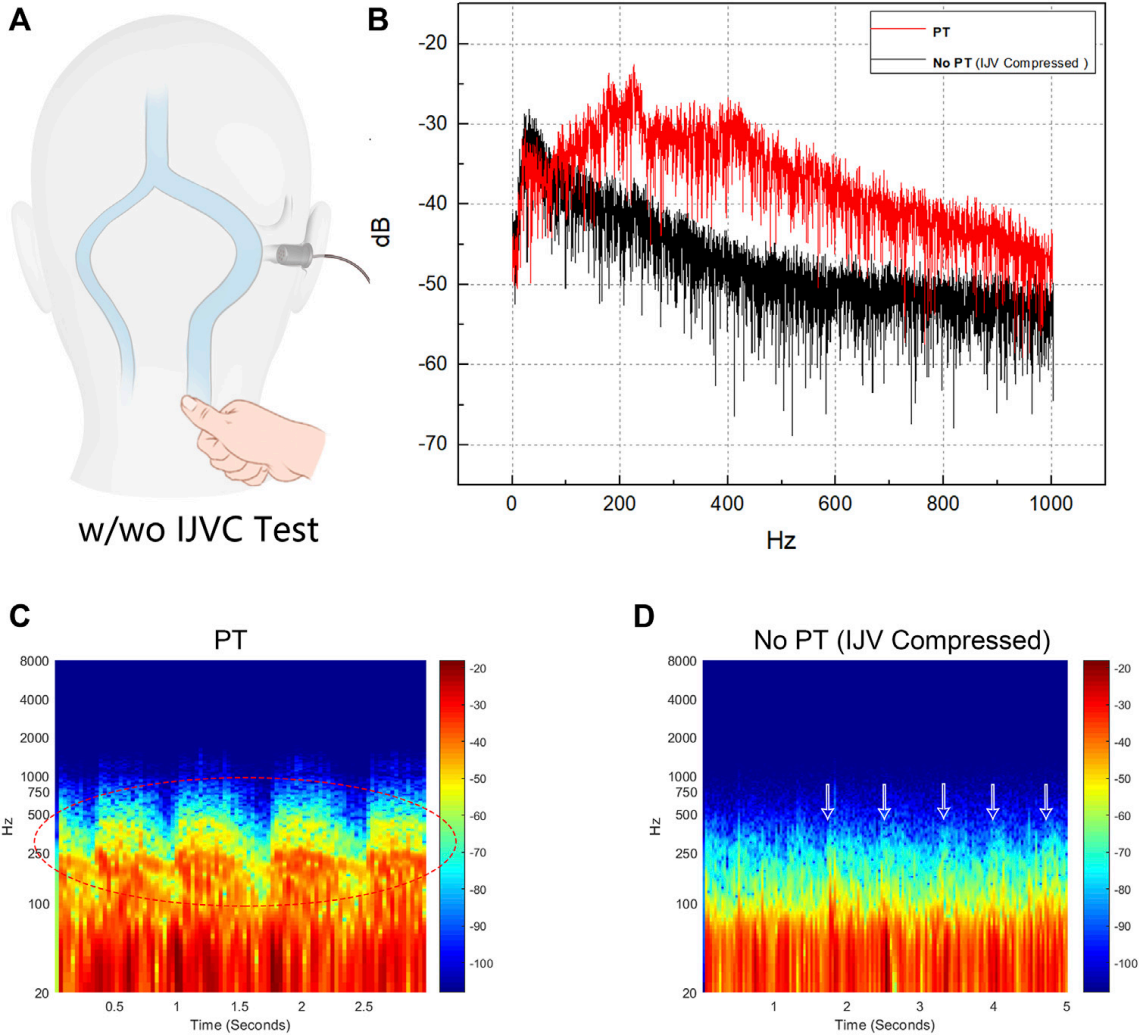
Results of trans-canal recording of produced pulse-synchronous somatosounds. PT indicates pulsatile tinnitus. **(A)** Schematic diagram of transcanal recording technique performed in junction with ipsilateral internal jugular vein (IJV) compression. **(B)** Frequency-amplitude diagram of the captured pulse-synchronous sound and without pulse-synchronous somatosound during which the ipsilateral IJV was compressed. **(C)** and **(D)** are the spectro-temporal results of the recorded pulse-synchronous somatosounds without IJV compression (PT) and with IJV compression (no PT). Notice that the main power of PT fluctuates below 1,000 Hz (red octave dashed lines) and PT disappeared despite that the low pulse-synchronous noises were still detectable (white arrows).

Results of spectro-temporal and frequency-amplitude results of the transcanal recording audio data are shown in Figure 10. The difference between the peak amplitude of PT with and without IJV compression (PT to no PT) revealed by transcanal recording was 10.3 dB (sensing range: 65–1,000 Hz). The frequency of peak amplitude of PT was 225.5 Hz. The difference between RMS amplitude of PT to no PT was 10.7 dB.

Results of the likeness rating for transcanal recording, Doppler hemoacoustics, CFD, and psychoacoustic frequency matching are shown in Table 4. The acoustic results are shown in Figure 8. The audio profile calculated by CFD was rated 8 out of 10, which was higher than that of the psychoacoustic method. In psychoacoustic testing, the given 125 Hz 1/3 octave narrow-band noise was rated the highest (score = 6). The overall likelihood of psychoacoustic testing was 6 out of 10. The loudness of the PT was matched at 25 dBSL in the contralateral ear.

**TABLE 4:** Results of subjective likeness ratings.

	Transcanal recording	Doppler ultrasound	Coupled CFD	Psychoacoustic matching
Subjective likeness (0–10)	10	9	8	6

## Discussion

This is the first coupled CFD study to crystallize the acoustic profile of both vibroacoustic/hydroacoustic sources of venous PT and displacement of the transverse-sigmoid sinus vascular wall by incorporating the setup of the sigmoid plate and dura mater into the settings of the boundary conditions. In addition, the current CFD acoustic outcomes were compared to the *in vivo* transcanal recording of PT combining the IJV compression test. Within the observed 20 from to 1,000 Hz in our CFD simulation, the peak and RMS amplitude of the vibroacoustic source were 1.49 and 3.3 times larger than those of the hydroacoustic noise production. However, it is noteworthy that the peak amplitude exhibited a large disparity among the probed regions of interest between the two acoustic sources. Because the wall pressure exerted on the vascular wall gives rise to vibroacoustic sound, the amplitude of the vibroacoustic noise diminishes as the wall pressure decreases gradually from the proximal to the distal end of the transverse-sigmoid sinus lumen. Therefore, for pressure-based coupled fluid-structure simulations where the force and displacement of fluid and solid interfaces are consistent, the loudness of the generated vibroacoustic noise predominantly depends on the magnitude of the exerted vascular wall pressure secondary to the physical flow impact. In this study, the results of the calculated vibroacoustic source near the transverse-sigmoid junction were comparable to those reported by Mu et al., where the simulated peak vibroacoustic noise generation fluctuates drastically between 111.5 and 65 dB (Mu et al., 2021a). Despite that they found the frequency of the main power of PT primarily situates above 1,000 Hz, considering the intrasubject heterogeneity in sinovenous hemodynamics, shapes/angulation of anatomical structures, and the current recorded PT sounds *in vivo* (Song et al., 2016; Hsieh et al., 2021b), we reckon that the results of this work are thus tenable.

The computational model of the dehiscent sigmoid plate reconstructed based on traditional CT images can also be inadequate for reproducing the structural domain for CFD simulations that conform to reality. Approximately 0.5 mm in total thickness of the osseous structure and dura mater can strictly restrict the magnitude of the vascular wall displacement down to the nanometer scale, and the displacement magnitude measured at the internal surface of the vascular wall underneath the dehiscence area was 9.6 times larger than that of the sigmoid plate. Based on these results, the generation of vibroacoustic noise may not be solely limited to the dehiscent area. This also explains why preclusion of vascular wall vibration using robust materials alone can be futile in eliminating PT in some subjects with SSWAs (Hsieh and Wang, 2020). The current simulated displacement outcome also conformed to our previous *in vivo* studies that failed to observe periodical vascular displacements below 0.72 microns before the separation of osseous and vascular walls (Hsieh et al., 2022b). Analogously, the vascular displacement gauged at vascular domains without the osseous attachment (brain tissue side) also exhibited a high accordance (at approximately 10-micron level) to previous *in vivo* findings acquired from the laser displacement sensor measurements (Hsieh et al., 2022a), which also matches the results reported by Mu et al. (2021b). Therefore, establishing a sigmoid plate and layers of dura mater in the boundary condition settings can impact the outcomes of CFD simulations and vibroacoustic sources and may greatly limit the displacement of vascular wall.

The hydroacoustic sound has rarely been investigated previously even though the blood flow noise (sometimes termed as turbulent/vortex sound) has long been deemed as one of the major factors causing PT (Otto et al., 2007). In this study, the current computed hydroacoustic amplitude was significantly lower than that measured by only, to our knowledge, *in vitro* experimental study using a hydrophone sensor (Hsieh et al., 2021a). We surmise that the fundamental difference derived from the insertion of the hydrophone sensor may impede flow patency and increase the downstream pressure gradient of the flow. It is notable that the hydroacoustic sound can also be detected using an electro-stethoscope, in which case the thickness and hardness of the 3D-printed materials may render collecting a lower amplitude (Valluru et al., 2020). The elbow-like sinus regions, such as the transverse-sigmoid sinus junction and jugular bulb, are typical locations where vortical flow structures emanate (Mu et al., 2022). The current CFD method demonstrated that the flow amplitude at the transverse sinus, transverse-sigmoid junction and jugular bulb was higher than that of the vascular segments without sharp turns, and these elbow-like regions exhibited a high-pressure gradient and/or flow velocity. In addition to vascular stenosis, which has a positive correlation with the pressure gradient (Zhao et al., 2021a; Zhao et al., 2021b; Ding et al., 2021), the entropic flow pattern of vortices generated in elbow-like regions may engender regional flow regurgitation that potentially compromises the patency of the sinus outflow (Zhao et al., 2021a), although the enigmatic correlation between the pressure gradient and vortex generation remains unraveled. Nevertheless, with growing evidence suggesting that the increased kinetic energy (sinus flow volume and velocity) is a primary contributing factor to PT rather than the occurrence of the vortex *per se*, it is postulated that the vortical structure of a flow contributes little or insignificantly to hydroacoustic noise production if the kinetic energy is relatively low (Gao et al., 2022).

The primary frequencies of the calculated vibroacoustic and hydroacoustic sounds were below 400 Hz. This finding conforms to the psychoacoustic loudness and frequency matching results, suggesting that the major power of the PT fluctuates mostly between the 200–600 Hz. However, the capability of obtaining accurate frequency components using the one-way fluid structure interaction analysis may be limited since the vascular wall may absorb certain frequency components (Liu et al., 2020). Although the peak RMS amplitude of the vibroacoustic noise calculated by the current CFD technique is more than four times larger than the objective loudness, given that approximately 10 dB reduction in the peak/RMS amplitude is required to silence PT, this specific range of amplitude reduction matches closely to the peak hydroacoustic A-weighted loudness for which a slight difference in the absolute value can be skewed by the degree of mastoid pneumatization and amplification (Tian et al., 2019b). Since the transmission loss of acoustic sources can occur and apply to both acoustic sources (Liu Z. H. et al., 2021), we extrapolate that both vibroacoustic and hydroacoustic noises both play a role in contributing the perception of PT, but the actual proportion remains clueless unless the minimal perceptive threshold, if present, is quantifiable. By incorporating the current clinical observations, PT may persist even after addressing dehiscence following transtemporal surgery. This suggests that in addition to the aerial transmission of sound inside the mastoid cavity, the bone-conduction route may be another but mostly overlooked transmission pathway of PT in some cases. Furthermore, emotional changes and/or cochlear sensitivity may also play a role in amplifying the subjective loudness of PT (Zhang et al., 2019). Thus, the subjective loudness of PT may potentially be affected by emotional conditions and/or bone-conduction sound transmission route that are yet to be discovered.

This study has five major limitations. First, blood is essentially a non-Newtonian fluid with shear-thinning effect. The non-Newtonian effect is significant in simulating wall shear stress but negligible for flow rate and pressure (Liu H. P. et al., 2021). Second, as this study focuses on the produced acoustic sources of flow and vascular wall motions, the transmission of PT sources regarding far field propagation inside the mastoid cavity were not discussed. However, building a reliable computational model of mastoid air cell compartments with the proper design of the mastoid pneumatization and intramastoid pressure, and assessing sound transmission from the middle to the inner ear can be extremely difficult, which will be discussed in future studies. In addition, the precise shape and uneven thickness of the harvested sigmoid plate structure in the dehiscent area render the computational design of a realistic sigmoid plate arduous. Third, although the results computed in this study acquired high subjective likeness, since CFD simulations rely heavily on the establishment of boundary conditions, the current one-way fluid-structure interaction setting ignored the internal blood flow pressure in response to the deformation of the venous wall, which can potentially hinder the acoustic outcomes. However, the lateral sinus wall lies against rigid osseous walls. Unlike arterial system, the wall of intracranial sinovenous conduit is less deformative and fixated by the intraluminal chordae willisii that restricts both dilation and compression of the venous walls, which, however, the further hemodynamic investigations are warranted. As we focused on calculating the low frequency range of the generated vibroacoustic and hydroacoustic sounds, frequency range above 1 kHz was omitted, thus creating a white-noise like in the vibroacoustic spectro-temporal results. However, we believe that the current simulated acoustic outcome closely conforms to the *in vivo* profile and participant’s subjective perception, which the current displacement and acoustic results are relatively compelling and assist in moving this research field a step forward. Fourth, another issue is the lack of a proper sound stimulus and reasonable frequency selection for psychoacoustic testing. The inability to modulate the rhythmic component of the given stimuli may reduce the likeness and misestimate the loudness of PT. Last, this study revealed the amplitude with and without the transmitted pulse-synchronous noise by utilizing the trans-canal recording. However, the microphone recording was uncalibrated, and the sound pressure level of the somatosound source was thus unknown. The source and transmittance/conversion of sound pressure into different media should be carefully investigated before acquiring reliable acoustic results, which further studies are warranted.

## Conclusion

The establishment of a thin osseous layer and dura mater overlying the sigmoid sinus vascular wall is commonly overlooked in CFD simulations of venous PT secondary to sigmoid plate dehiscence. The displacement of the vascular wall underneath the dehiscent area was 9.6 times larger than that of the outermost sigmoid plate, while it was 3,617 times smaller than that across the vascular wall away from the surfacing osseous structures. Macroscopically, as the sinovenous wall pressure decreases from upstream to downstream, the amplitude of the produced vibroacoustic noise descents from the proximal to distal end of the transverse-sigmoid sinus. These computed results suggest that the source of vibroacoustic noise may derive from forced vibrations of the entire transverse-sigmoid sinus vascular tissue caused by the sinus flow impact, which is not limited to the focal dehiscent area. On the other hand, the amplitude of hydroacoustic noise correlates to the magnitude of pressure gradient and flow velocity, in which regions with stenoses and sharp sinus angulations contribute to the production of hydroacoustic sounds. By incorporating the transcanal recording technique with IJV compression test, the primary frequency of PT was found fluctuating below 1,000 Hz, which aligns with the trend provided by the current coupled simulation technique; however, the peak amplitude of *in vivo* pulse-synchronous noise measures approximately 10 dB (matching PT on-and-off subjectively). This value is comparatively lesser than both calculated vibroacoustic/hydroacoustic acoustic sources and the perceived loudness of PT. Unless the 1) air-conduction and/or bone-conduction transmission pathways, 2) the effect of intramastoid acoustic impedance/amplification, and 3) the perceptive threshold of PT are fully clarified, further refinements on sensing and computational applications are warranted to provide useful details on surgical optimization/customization and minimize the incidence of surgical failure regarding both transtemporal/endovascular approaches.

## Data Availability

The original contributions presented in the study are included in the article/Supplementary Material, further inquiries can be directed to the corresponding author.

## Supplementary material

SUPPLEMENTARY FILE S1 Audio outcome simulated by the computational fluid dynamics.

SUPPLEMENTARY FILE S2 Audio data of pulsatile tinnitus captured using the trans-canal recording technique.

SUPPLEMENTARY FILE S3 Audio data of pulsatile tinnitus suppressed by ipsilateral internal jugular vein compression.

SUPPLEMENTARY FILE S4 Audio data of the recorded Doppler ultrasound vascular sound.
